# Urinary Bladder Adenocarcinoma Metastatic to the Abdominal Wall: Report of a Case with Cytohistologic Correlation

**DOI:** 10.1155/2016/8608412

**Published:** 2016-02-23

**Authors:** Vikas Nath, Mithra Baliga

**Affiliations:** Department of Pathology, University of Mississippi Medical Center, 2500 North State Street, Jackson, MS 39216, USA

## Abstract

We report a case of adenocarcinoma metastatic to the abdominal wall in a 71-year-old man with a history of primary bladder adenocarcinoma. CT-guided core biopsy was performed; imprints and histologic sections showed malignant glands lined by tumor cells with hyperchromatic nuclei and prominent nucleoli, infiltrating through skeletal muscle. Immunohistochemistry revealed positivity for CK7, membranous/cytoplasmic *β*-catenin, caudal-type homeobox transcription factor 2 (CDX2), and *α*-methylacyl coenzyme A racemase and negativity for CK20, p63, prostate-specific antigen (PSA), and prostate-specific acid phosphatase (PSAP). These findings were interpreted as metastatic adenocarcinoma, consistent with bladder primary. Primary bladder adenocarcinoma is a rare malignancy arising within glandular metaplasia and is associated with cystitis cystica and cystitis glandularis. Predisposing factors include bladder exstrophy, schistosomiasis, and other causes of chronic bladder irritation. This tumor is divided into intestinal, clear cell, and signet ring cell subtypes. Treatment involves radical cystectomy with pelvic lymph node dissection, and prognosis is unfavorable. Primary bladder adenocarcinoma should be differentiated from urachal adenocarcinoma, which arises from urachal remnants near the bladder dome, and secondary adenocarcinoma, or vesical involvement by adenocarcinoma from a different primary. CK7, CK20, CDX2, thrombomodulin, and *β*-catenin can help distinguish primary bladder adenocarcinoma from colonic adenocarcinoma; PSA and PSAP can help distinguish primary bladder adenocarcinoma from prostate adenocarcinoma.

## 1. Introduction

Primary adenocarcinoma of the urinary bladder is an uncommon malignancy that accounts for 0.5 to 2% of all malignant bladder neoplasms [1–5]; it is the third most common type of bladder carcinoma, after urothelial carcinoma (UC) and squamous cell carcinoma [1, 2]. Most often, it presents in the sixth decade of life and has a predilection for males [1, 3, 6]. Presenting symptoms typically include hematuria, urinary retention, and irritation while voiding [1, 3, 7]; mucus in the urine is a hallmark of adenocarcinoma, though it is not always present [1, 8]. Bladder adenocarcinoma is associated with a variety of irritative and inflammatory conditions. It is the most common type of bladder carcinoma that arises in patients with exstrophy of the bladder; indeed, these patients have a 4% lifetime risk of developing this particular carcinoma. Schistosomiasis, endometriosis, and bladder augmentation have also been mentioned as predisposing factors [1, 3, 4].

Before rendering a diagnosis of primary adenocarcinoma, it is important first to exclude the other 2 types of adenocarcinomas that can occur in the bladder, urachal adenocarcinoma and secondary adenocarcinoma; the latter consists of neoplasms that do not originate from the bladder but involve it either by local extension or by lymphatic or hematogenous metastasis [1, 2]. Local extension is most commonly by malignancies of the colon, prostate, and female genital tract; metastasis is most commonly from the stomach, breast, and lung [2, 3, 5]. We report a patient with primary bladder adenocarcinoma who developed a metastasis to the abdominal wall, diagnosed by examination of imprints and histologic sections of a CT-guided core biopsy.

## 2. Case Report

A 71-year-old African-American male presented at an outside hospital with acute renal failure and bilateral hydronephrosis and was found to have bladder adenocarcinoma with invasion of the muscularis propria. His past medical history was significant for nephrolithiasis, prostate adenocarcinoma treated with external beam radiation and bilateral simple orchiectomy 20 years previously, and transurethral bladder resection with bilateral ureteral stent placement at an outside hospital. The patient was a poor historian, and no medical records could be obtained documenting either the histology of his bladder and prostate tumors or the known extent of his disease or the diagnostic and therapeutic procedures undertaken at the outside hospital. He was referred to our institution for evaluation for possible cystoprostatectomy with colonic conduit. A preoperative colonoscopy showed a tubular adenoma but was otherwise negative, and his serum prostate-specific antigen (PSA) level was less than 0.01 ng/mL.

**Figure 1:**
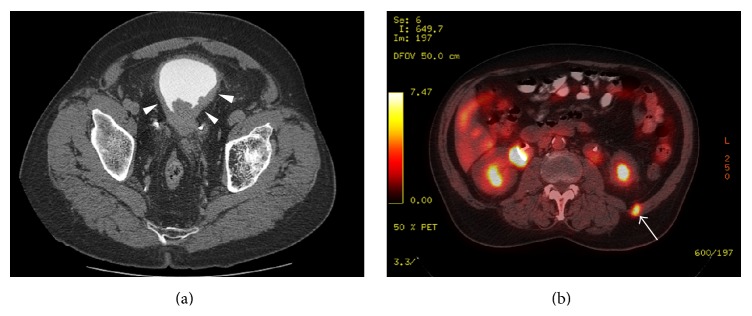
Imaging studies: (a) CT of the abdomen and pelvis showing diffuse bladder wall thickening (*arrowheads*) with perivesical fat stranding, indicating involvement of the bladder by invasive carcinoma; (b) PET of the abdomen and pelvis showing a 1 cm FDG-avid nodule in the musculature of the left flank, suspicious for metastasis (*arrow*).

The patient was scheduled for surgery, but this was canceled after he underwent CT of the abdomen and pelvis, which revealed pulmonary nodules measuring up to 0.6 cm in diameter in the right upper lobe, left upper lobe, and lingula that were suspicious for metastases. In addition, the CT revealed retroperitoneal adenopathy of the left paraortic and aortocaval lymph nodes measuring up to 1.9 cm in diameter; bilateral hydronephrosis and hydroureter; and diffuse bladder wall thickening with perivesical soft tissue stranding, indicative of invasion by a bladder tumor (Figure 1(a)). Multiple filling defects were present in the bladder lumen, which were also interpreted as secondary to invasive carcinoma. Positron emission tomography (PET) with an ^18^F-fluorodeoxyglucose (FDG) tracer showed no abnormal metabolic activity in either the pulmonary or the retroperitoneal masses; however, FDG avidity was observed in a left cervical lymph node and in a 1 cm nodule within the abdominal wall musculature of the left flank (Figure 1(b)). The primary tumor, at the base of the bladder, measured 11.7 cm in greatest dimension; it also showed hypermetabolic activity and appeared to invade into the prostate.

**Figure 2:**
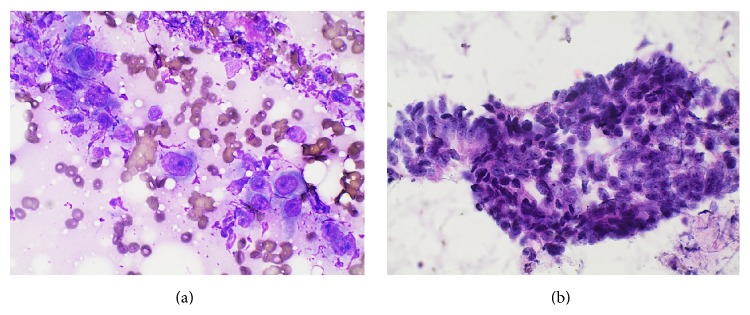
Imprints of the CT-guided core biopsy specimen from the left flank mass showing clusters of malignant columnar cells with a high nuclear/cytoplasmic ratio and prominent nucleoli: (a) Diff-Quik and (b) hematoxylin and eosin, ×600.

**Figure 3:**
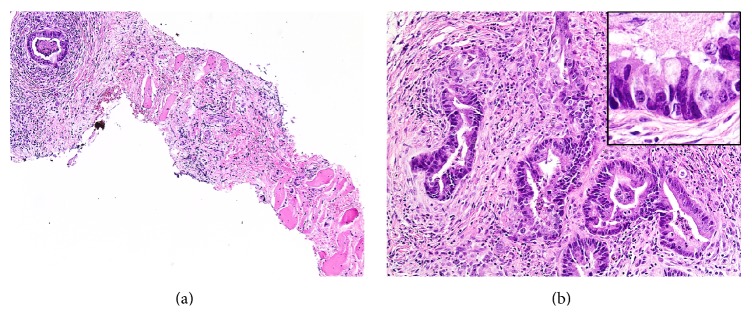
Histologic sections of the biopsy. (a) Malignant glands are seen infiltrating through skeletal muscle (hematoxylin/eosin, ×100). (b) The glands are lined by columnar cells with intestinal epithelial morphology and contain necrotic debris in the lumina (hematoxylin and eosin, ×200); occasional goblet cells are also present (*inset*, hematoxylin and eosin ×600).

**Figure 4:**
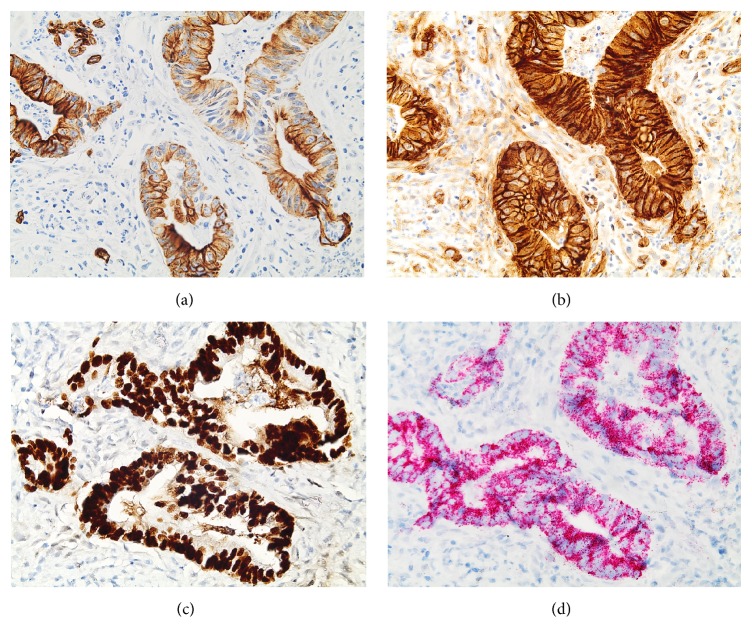
IHC of the biopsy showing positive expression of (a) CK7, (b) *β*-catenin in a membranous/cytoplasmic staining pattern, (c) CDX2, and (d) AMACR (×400).

Ultrasound-guided fine needle aspiration was attempted on the cervical lymph node, but insufficient tissue was obtained, so CT-guided core biopsy was performed on the abdominal wall mass. Imprints of the biopsy specimen showed crowded clusters of tumor cells with high nuclear/cytoplasmic ratios and prominent, small nucleoli (Figure 2). Histologic sections of the biopsy showed malignant glands lined by intestinal type columnar cells and occasional goblet cells, with necrotic debris in the lumina, infiltrating through skeletal muscle (Figure 3). The glands were surrounded by desmoplastic stroma and acute and chronic inflammatory cells. Immunohistochemistry (IHC) revealed that the malignant glands were positive for CK7, *β*-catenin (membranous/cytoplasmic staining pattern), caudal-type homeobox transcription factor 2 (CDX2), and *α*-methylacyl coenzyme A racemase (AMACR) (Figures 4(a)–4(d)) and negative for CK20, p63, PSA, and prostate-specific acid phosphatase (PSAP). These findings were interpreted as being consistent with metastatic adenocarcinoma; given that the patient had a concurrent vesical tumor, his bladder adenocarcinoma was considered the primary site. Colonic adenocarcinoma with local extension to the bladder was deemed a less likely diagnosis, since there was no evidence of a primary colonic tumor; however, prostate adenocarcinoma with local extension was a consideration given the patient's past medical history. Neither of these possibilities could be excluded based solely on histology and IHC.

In lieu of surgery, the patient was scheduled for 8 cycles of gemcitabine/cisplatin. After completing 4 cycles, he experienced renal failure and was admitted to an outside hospital in Vicksburg, MS, where he underwent exchange of his ureteral stents and antibiotic therapy for purulent cystitis; chemotherapy was withheld while this treatment was taking place. Although repeat CT and PET scans initially showed that his disease was stable, a CT performed 3 months after his last cycle of chemotherapy showed bilateral hydroureteronephrosis, heterogeneous enhancement of the left kidney with perinephric stranding and fascial thickening, and circumferential thickening of the wall of the left ureter with periureteral fat stranding. A soft tissue mass was identified in the hilum of the left kidney, suggesting a renal hilar neoplasm with superimposed pyelonephritis and ureteritis, and the patient was admitted and given intravenous antibiotics. Urine culture from an earlier clinic visit grew coagulase-negative* Staphylococcus*; however, inpatient cultures were negative. Urology was consulted, and based on his symptoms it was determined that he most likely did not have pyelonephritis, as he lacked the characteristic clinical triad of flank pain, leukocytosis, and fever. He was discharged home in stable condition.

Eleven days later, the patient presented to an outside hospital with urosepsis, renal failure, and hypotension and was found by CT to have right-sided hydronephrosis and a hemorrhagic left renal mass, in keeping with previous imaging findings at our institution. A nephrostomy tube was placed in the right kidney. Cystoscopy was performed, and tissue was obtained from the bladder mass that was interpreted as moderately differentiated, partially necrotic adenocarcinoma. The tumor was positive for cytoplasmic *β*-catenin; nuclear staining could not be assessed. CDX2 and villin immunostains were also positive; thrombomodulin was positive in tumor vessels but not within the tumor cells themselves. These findings were consistent with the staining pattern we observed in the metastatic tumor tissue. During the course of the patient's hospital stay, his renal function and hypotension continued to worsen, leading to cardiopulmonary arrest. Despite the resuscitative efforts, he ultimately expired eight days following admission.

## 3. Discussion

### 3.1. Primary Adenocarcinoma

Primary adenocarcinoma of the urinary bladder is derived from urothelium that has undergone glandular metaplasia, often in the context of chronic irritation of the vesical mucosa. Metaplasia of cystitis cystica by mucin-producing columnar cells results in cystitis glandularis, which is considered the precursor lesion of primary adenocarcinoma [1, 4]. Glandular metaplasia may also occur without urothelial invagination, resulting in a variable cystoscopic appearance: sessile, papillary, nodular, or ulcerated [1, 3]. Of the various subtypes described in the literature, the intestinal, clear cell, and signet ring cell subtypes are the best known [2, 9, 10].

Intestinal type adenocarcinoma is the most common of the 3 subtypes [9, 11]. Its cytomorphology is characterized by crowded clusters of columnar or cuboidal cells resembling colonic epithelial cells, which may show pleomorphism, with hyperchromatic nuclei and vacuolated cytoplasm. Mucus is often present in the background [2, 9–11]. On histology, infiltrating glands are seen that are lined by tall columnar cells virtually identical to their colonic counterparts, surrounded by desmoplastic stroma. Luminal necrosis, extracellular mucin, and cystitis cystica in the adjacent urothelium may also be present [3, 5].

Clear cell adenocarcinoma is more common in females than males and often originates in or near the urethra [3, 9, 10, 12]. Cytologic specimens are composed of abundant cells in round clusters that have enlarged nuclei, thickened nuclear membranes, prominent nucleoli, and mitoses. The cytoplasm is abundant, vacuolated, and clear due to glycogenation [7, 9–12]. Acute inflammatory cells may be present either in the background or within intracytoplasmic vacuoles [7, 9, 11, 12]. Histologic exam shows a tumor with tubular, solid, or papillary architecture composed of cuboidal to columnar cells. There is usually marked atypia, and a dense inflammatory infiltrate is often present [3, 7, 12].

Signet ring cell adenocarcinoma has a dismal prognosis, with 5-year survival, a mere 23% [1, 4, 13]. This diagnosis requires that signet ring cells comprise the tumor exclusively, as these cells may also be components of mixed adenocarcinomas or UC [9, 11, 13]. Grossly, these tumors are infiltrative, with subepithelial growth that results in bladder wall thickening; often, no discrete mass is visible on cystoscopy [3, 4]. Cytologic specimens consist of small, dissociated cells with large mucin-filled cytoplasmic vacuoles that displace the nucleus; pools of mucin are seen in the background [5, 9, 11]. A combination of periodic acid-Schiff (PAS), cytokeratin, and CD45 can help to distinguish this tumor from lymphoma with a signet ring cell appearance [4, 9, 13]. Cytologic specimens must be evaluated for other types of malignant cells, such as urothelial cells, to exclude a nonbladder primary before this diagnosis can be made [13]. The histologic appearance is that of sheets of signet ring cells infiltrating the lamina propria and muscularis [3].

Though the majority of tumors occur near the trigone [2, 3], primary adenocarcinoma may arise anywhere in the bladder and may be either unifocal or multifocal [1, 3]. Most tumors show detrusor muscle invasion at the time of diagnosis [1, 6]. Treatment is surgical and consists of radical cystectomy with pelvic lymph node dissection [3, 4, 8]. Chemotherapy is of questionable value. Cisplatin is considered first-line therapy for UC [8], and 5-fluorouracil is effective in colorectal carcinoma, but neither has been shown to be of much value in primary adenocarcinoma [1, 4]. Clinical stage is the most important prognostic factor in this disease [3, 8], and survival is generally poor, ranging from 27% to 61% [4]. Although females are less commonly affected, their prognosis appears poorer than that of males, and their presentation is at a higher stage [6].

### 3.2. Urachal Adenocarcinoma

Urachal adenocarcinoma is believed to be derived from urachal remnants [5, 14, 15]. Cytologic features are variable; specimens may be composed of either sheets of columnar cells with vacuolated cytoplasm and mild nuclear membrane irregularity [14] or pleomorphic cells arranged in papillae and acini with prominent nucleoli and abundant mitoses [15]. Intracytoplasmic and/or extracellular mucin is often present and stains with PAS [14, 15]. Histologic examination reveals tumor cells floating in pools of extracellular mucin, which may have columnar or signet ring cell morphology [5].

Among the criteria [3–5, 8] used to diagnose bladder adenocarcinoma as urachal in origin are (1) location at the bladder dome; (2) sharp demarcation between the tumor and surface epithelium; and (3) absence of another primary site. As urachal adenocarcinoma typically originates in the anterior wall or dome of the bladder, the standard treatment involves partial cystectomy of the dome as part of an* en bloc* resection [1, 4, 5, 8]. The urothelium adjacent to the tumor is usually devoid of adenocarcinoma* in situ* or cystitis cystica/glandularis, unlike primary adenocarcinoma [4, 14, 15]. IHC reveals diffuse positivity for the high-molecular weight cytokeratin 34*β*E12 and membranous, but not nuclear, expression of *β*-catenin. These features are helpful in differentiating urachal from colonic adenocarcinoma, as both of these tumors are known to express CK20 and CDX2 [3, 5, 14, 15].

### 3.3. Secondary Adenocarcinoma

Secondary adenocarcinoma is more common than primary adenocarcinoma, and differentiating the two in cytologic preparations can be difficult. Colonic adenocarcinoma is characterized by pleomorphic cuboidal to columnar cells, sometimes arranged in glands, with vacuolated cytoplasm, irregular nuclear borders, and prominent nucleoli. “Dirty” necrosis consisting of cytoplasmic and nuclear debris is often present in the background [2]. Histologically, it is virtually indistinguishable from the intestinal type of primary bladder adenocarcinoma. IHC can be helpful, as bladder adenocarcinoma is positive for CK7, CK20, CDX2, and thrombomodulin and negative for nuclear *β*-catenin. By contrast, colonic adenocarcinoma, while positive for CK20 and CDX2, is negative for CK7 and positive for thrombomodulin and nuclear *β*-catenin [3, 5]. Thrombomodulin is a highly specific marker for bladder adenocarcinoma, but it is expressed in only 59% of these tumors. Cytokeratins must also be interpreted with caution, as occasional colonic carcinomas may be CK7 positive; in addition, 29% of bladder adenocarcinomas are CK7 negative and CK20 positive, a profile that matches that of most colonic adenocarcinomas [3, 5].

Another tumor that can invade the bladder by local extension and mimic primary bladder adenocarcinoma is prostate adenocarcinoma, in particular prostatic ductal carcinoma. This rare tumor features cells in sheets or papillary fronds with abundant granular cytoplasm, bland nuclei showing enlargement and hyperchromasia, and coarse chromatin with prominent nucleoli and variable mitoses [16–18]. Histologic sections show malignant glands in a variety of architectural patterns including papillary, cribriform, and acinar, depending on whether the tumor originates from primary or secondary prostatic ducts [16, 17]. Prostate adenocarcinoma may be readily differentiated from bladder adenocarcinoma by virtue of its positivity for PSA and PSAP [5, 16–18]; other markers that help to confirm a prostate origin include Leu7 and AMACR [5, 16].

## 4. Conclusion

The diversity of glandular neoplasms that can occur in the urinary bladder makes the diagnosis of primary bladder adenocarcinoma in cytologic preparations uniquely challenging. In our patient's case, the lesion sampled was a metastasis from his bladder tumor, which meant that the knowledge of his past medical history and imaging findings was crucial in generating a differential diagnosis and choosing the appropriate immunostains. The positivity of the tumor for CK7, *β*-catenin in a membranous/cytoplasmic staining pattern, CDX2, and AMACR, and negativity for CK20, p63, PSA, and PSAP, favored a vesical rather than a colorectal or prostatic origin. Colonic adenocarcinoma typically expresses CDX2, but it is negative for CK7 and positive for CK20 and expresses *β*-catenin in a nuclear pattern. Likewise, prostate carcinoma expresses PSA and PSAP. We were careful not to interpret the IHC findings as unequivocal evidence of vesical origin, as they were not specific for primary bladder adenocarcinoma. Secondary adenocarcinoma, being more common, had to be excluded first, and this was not possible to do.

The IHC results do not exclude urachal adenocarcinoma either. However, this was not a distinction we attempted to make, not only on account of the advanced stage of the patient's disease, but also because of the urachal type adenocarcinoma that is a diagnosis better made on histologic sections to assess the condition of the urothelium and to note the presence of urothelial remnants. Primary adenocarcinoma of the urinary bladder is not frequently encountered in the realm of cytology, and the differential diagnosis includes a variety of neoplasms, not all of which originate from the vesical mucosa. Evaluation of cytologic specimens in which this malignancy is suspected requires correlation with the patient's clinical history, imaging, IHC, and, ideally, histologic assessment of bladder biopsy or cystectomy specimens to eliminate other primary sources and establish the tumor's vesical origin.
